# Direct comparison uptake patterns of ^99m^Tc-PYP and ^99m^Tc-HMDP scintigraphy in cardiac amyloidosis with semi-quantitative analysis

**DOI:** 10.1007/s11604-026-01978-8

**Published:** 2026-03-19

**Authors:** Toshiya Ensako, Takashi Norikane, Yuka Yamamoto, Yasukage Takami, Yuri Manabe, Mitsumasa Murao, Masashi Imajo, Katsuya Mitamura, Keigo Omori, Akihiro Oishi, Masatoshi Morimoto, Takahisa Noma, Yoshihiro Nishiyama

**Affiliations:** 1 Department of Radiology, Faculty of Medicine, Kagawa University, 1750-1 Ikenobe, Miki-Cho, Kita-Gun, Kagawa, 761-0793 Japan; 2 Division of Clinical Radiology, Department of Medical Technology, Kagawa University Hospital, Kagawa, Japan; 3 Department of Cardiorenal and Cerebrovascular Medicine, Faculty of Medicine, Kagawa University, Kagawa, Japan

**Keywords:** Cardiac amyloidosis, ATTR, Scintigraphy, PYP, HMDP

## Abstract

**Background:**

Bone-avid tracers, such as ^99m^Tc-pyrophosphate (PYP) and ^99m^Tc-hydroxymethylene diphosphonate (HMDP), facilitate the noninvasive diagnosis of transthyretin cardiac amyloidosis (ATTR-CA). In this study, we compared these two methods.

**Methods:**

We retrospectively reviewed 83 patients with suspected or diagnosed cardiac amyloidosis (mean age, 79 years; 11 women) who underwent PYP and HMDP scintigraphy. Early (1 h) imaging was available for of 72/50 patients with PYP/HMDP, and all patients underwent delayed (3 h) imaging. Visual grading (Dorbala score), heart-to-contralateral lung (H/CL) ratios, and relative segmental uptake (RSU) derived from SPECT (3 h) polar map analysis using the quantitative perfusion SPECT software were compared.

**Results:**

visual scores showed substantial agreement. The H/CL ratio showed a strong correlation. Bland–Altman analysis revealed fixed and proportional biases, with discrepancies increasing at higher uptake levels. The median H/CL values were comparable at 1 h, whereas HMDP showed slightly higher delayed values. Receiver operating characteristic analysis indicated excellent and good diagnostic accuracy at 1 and 3 h, respectively. Using routine diagnostic criteria, SPECT visual assessment demonstrated comparable diagnostic performance between PYP and HMDP. RSU analysis in 34 patients demonstrated similar segmental uptake distributions for both tracers, with basal and septal predominance, consistent with the apical-sparing patterns described previously.

**Conclusions:**

PYP and HMDP demonstrated comparable diagnostic accuracies for ATTR-CA. PYP has been extensively validated and is suitable for early imaging, whereas HMDP has a slightly higher delayed uptake and lower blood pool activity. When interpreted using routine diagnostic criteria, SPECT provided equivalent diagnostic performance for PYP and HMDP. Segmental analyses confirmed the preservation of the characteristic apical sparing across the tracers. Both tracers are clinically interchangeable when standardized acquisition and interpretation protocols are applied.

## Introduction

Cardiac amyloidosis (CA) is an infiltrative cardiomyopathy caused by the extracellular deposition of misfolded amyloid fibrils, most commonly of the transthyretin (ATTR) or immunoglobulin light-chain (AL) type. Accurate differentiation between transthyretin cardiac amyloidosis (ATTR-CA) and immunoglobulin light-chain cardiac amyloidosis (AL-CA) is clinically essential because their therapeutic strategies, prognoses, and monitoring differ substantially. Over the last decade, advances in nuclear cardiology have established bone-avid tracers labeled with technetium, such as ^99m^Tc-pyrophosphate (PYP), ^99m^Tc-hydroxymethylene diphosphonate (HMDP), and ^99m^Tc-3,3-diphosphono-1,2-propanodicarboxylic acid (DPD), as noninvasive diagnostic tools for ATTR-CA.

Major guidelines in Japan, the United States, and Europe endorse the use of these tracers. The Japanese Circulation Society (JCS) 2020 incorporated PYP imaging into the diagnostic algorithm for systemic ATTR amyloidosis, recommending visual (Perugini grade) and semi-quantitative heart-to-contralateral lung (H/CL) ratio assessments to support a biopsy-free diagnosis when AL amyloidosis has been excluded [1].

In the United States, the American Society of Nuclear Cardiology (ASNC) published practice points emphasizing a standardized protocol for PYP acquisition (planar and single photon emission computed tomography/ computed tomography (SPECT/CT)), visual grading of rib uptake, and semiquantitative H/CL ratios [2]. The 2023 American College of Cardiology Expert Consensus further reinforces the central role of bone scintigraphy in the multidisciplinary care of patients with CA [3]. In Europe, the European Society of Cardiology 2021 position statement and joint ASNC/European Association of Nuclear Medicine documents endorsed PYP, DPD, and HMDP as equivalent tracers for the non-invasive diagnosis of ATTR-CA, with SPECT/CT recommending distinguishing between true myocardial uptake from the blood pool activity and the adjacent bone [4, 5].

The current diagnostic approach is supported by landmark clinical evidence that bone tracer scintigraphy, when combined with the exclusion of AL-CA, allows a non-biopsy diagnosis of ATTR-CA with high specificity [6]. In addition, standardization efforts by international societies have emphasized the need for harmonization of acquisition protocols and interpretation criteria across tracers, including PYP, DPD, and HMDP, to ensure the consistency and reproducibility of imaging results [7]. The Perugini scoring system, originally developed using DPD scintigraphy, remains the basis for the contemporary visual assessment of cardiac uptake [8].

While most validation studies have historically focused on PYP and DPD, accumulating evidence indicates that HMDP is highly reliable for ATTR-CA imaging and is more widely available in Japan and parts of Europe. Recent single-center analyses have confirmed the diagnostic utility of HMDP, including its role in monitoring the response to tafamidis treatment [9]. Furthermore, multi-institutional Japanese working-group reports have highlighted the importance of harmonizing imaging protocols for both PYP and HMDP in clinical practice [10].

Despite the broad endorsement of these guidelines and growing clinical experience, direct comparative evidence between PYP and HMDP, particularly regarding uptake patterns, semi-quantitative measures, and reproducibility, remains limited. Therefore, further evaluation is warranted to optimize tracer selection and standardize diagnostic criteria across institutions, thereby ensuring consistent and guideline-concordant care for patients with suspected cardiac amyloidosis.

## Material and method

### Study design and patients

This retrospective, single-center study enrolled 83 consecutive patients who underwent both PYP and HMDP scintigraphy for the diagnosis of suspected cardiac amyloidosis between March 2018 and December 2024. At our institution, both tracers may be used in routine clinical practice at the discretion of the treating physicians when clinically indicated to improve diagnostic confidence. No imaging studies were performed specifically for research purposes. The study population comprised 11 women aged 60–94 years (median, 79 years). The study population comprised 83 patients (11 women) aged 60–94 years (median, 79 years). The majority of patients were referred for scintigraphy because cardiac amyloidosis was suspected based on a prior cardiac evaluation, primarily transthoracic echocardiography. Left ventricular hypertrophy was present in 77 patients and heart failure with preserved ejection fraction was observed in 64 patients. No patient with severe aortic stenosis was included. The interval between the PYP and HMDP examinations was 3–35 days (median, 7 days). PYP scintigraphy was performed in 36 patients and HMDP scintigraphy was performed first in 47 patients.

According to the diagnostic criteria of the JCS 2020 guidelines, 77, 4, and 2 patients were diagnosed with ATTR-CA, AL-CA, and non-amyloidosis, respectively.

The study protocol was approved by the institutional review board of the host institution and the requirement for written informed consent was waived because of its retrospective design.

### Imaging protocol

All patients received 740 MBq of PYP (PD Radiopharma Inc., Tokyo, Japan) or HMDP (Nihon Medi-Physics, Tokyo, Japan) intravenously. For PYP scintigraphy, early (1-h) and delayed (3-h) planar images were obtained from 72 patients, whereas only 11 patients underwent delayed imaging. For HMDP, early and delayed acquisitions were performed in 50 patients, whereas only 33 patients underwent delayed imaging.

Planar images were acquired in the anteroposterior view by using a dual-head gamma camera (Symbia T16; Siemens Healthineers, Erlangen, Germany) equipped with a low-energy high-resolution (LEHR) collimator. The acquisition energy window was centered at 140 keV, with a ± 20% width.

SPECT/CT imaging was performed 3 h after the tracer injection for both PYP and HMDP. For PYP, SPECT images were acquired in the continuous mode with 20 s per view over 36 views, resulting in a total acquisition time of 12 min. For HMDP, SPECT images were acquired in the step-and-shoot mode with 40 s per view over 30 views for a total acquisition time of 20 min. These acquisition parameters were applied consistently for the quantitative analysis. All systems were equipped with LEHR collimators and configured with an energy window centered at 140 keV ± 21%. Image reconstruction was performed using an ordered subset expectation–maximization algorithm with 15 iterations and six subsets. A Gaussian post-reconstruction filter with an 11-mm full width at half maximum was uniformly applied across all examinations to standardize the image quality.

### Image analysis

Planar images were visually graded using the Dorbala visual scoring system (0–3) and semi-quantitatively analyzed using the heart-to-contralateral lung ratio (H/CL) according to ASNC guidelines [2, 7, 11]. A correlation analysis of the H/CL values between the PYP and HMDP data was performed for both early (1-h) and delayed (3-h) imaging.

SPECT images were analyzed using quantitative perfusion SPECT (QPS) software (Cedars-Sinai Medical Center, Los Angeles, CA, USA) as an image analysis tool to generate LV segmentation and polar maps; no myocardial perfusion SPECT was performed. Patients in whom heterogeneous uptake or excessive extra-cardiac accumulation precluded reliable polar map generation were excluded, leaving 34 evaluable patients. To characterize the myocardial uptake distribution patterns, we defined the relative segmental uptake (RSU) as the mean count of each American Heart Association (AHA) 17-segment divided by the mean count of the entire LV myocardium. This normalization was chosen to reduce dependence on a single maximum uptake segment (which can dominate conventional relative scaling) and to better reflect regional distribution patterns.

All analyses were conducted by a board-certified nuclear cardiologist with > 15 years of experience in cardiac nuclear medicine (Takashi, N.). In cases where visual interpretation was challenging, the final judgment was made by consensus among the co-authors, all of whom were experienced nuclear medicine physicians.

### Statistical analysis

All analyses involved direct comparisons of PYP and HMDP across the entire cohort. Agreement in visual grades between tracers was evaluated using Cohen’s kappa (reported for 1 and 3 h). Continuous data are presented as the mean ± SD or median (IQR) per Shapiro–Wilk. Paired comparisons between the tracers were performed using paired t-tests or Wilcoxon signed-rank tests, as appropriate.

Associations between the tracers were determined using Spearman’s rank correlation (H/CL at 1 and 3 h; segment-level RSU). Bland–Altman analyses assessed agreement and tested for fixed and proportional biases at 1 and 3 h. Interobserver reproducibility testing was not performed. For clinically relevant diagnostic evaluations, additional analyses were performed using the established cutoff values. For visual assessment, a cutoff of visual grade ≥ 2 was applied. For planar semi-quantitative analysis, H/CL cutoff values of ≥ 1.5 at 1 h and ≥ 1.3 at 3 h imaging were used. Diagnostic performance was evaluated by calculating the sensitivity, specificity, and overall diagnostic accuracy. Receiver operating characteristic (ROC) curve analysis was conducted to assess the diagnostic performance of the planar and tomographic parameters, and the area under the curve (AUC) was calculated. Two-sided *p* < 0.05was considered significant. All statistical analyses were performed using SPSS version 30 (IBM Corp., Armonk, NY, USA). 

## Results

### Planar imaging: visual analysis (Table 1)

**Table 1:** Cross-tabulation of Dorbala visual scores obtained with PYP and HMDP at 3 h

HMDP	Score 0	Score 1	Score 2	Score 3	total
PYP					
**PYP and HMDP at 1 h**					
Score 0	0	0	0	0	0
Score 1	0	1	0	1	2
Score 2	0	2	5	0	7
Score 3	0	0	4	35	39
total	0	3	9	36	48
**PYP and HMDP at 3 h**					
Score 0	0	0	0	0	0
Score 1	6	5	1	0	12
Score 2	2	4	10	2	18
Score 3	0	1	8	44	53
total	8	10	19	46	83

The table demonstrates concordance between tracers in the visual grading of myocardial uptake

PYP: pyrophosphate; HMDP: hydroxymethylene diphosphonate

HMDP: ^99m^Tc-hydroxymethylene diphosphonate; PYP: ^99m^Tc-pyrophosphate

**Fig. 1:**
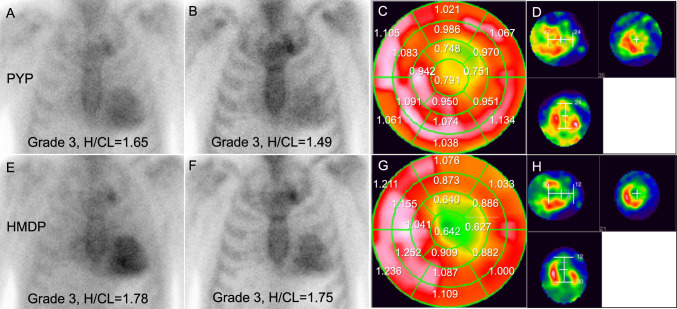
Representative case of a man in his 70 s with transthyretin cardiac amyloidosis. Upper row (**A**–**D**): PYP; lower row (**E**–**H**): HMDP. From left to right: planar images at 1 h, planar images at 3 h, polar maps, and representative SPECT slices. Both tracers demonstrated intense myocardial uptake. Polar maps (**C**, **G**) display relative segmental uptake (RSU) values. *PYP: pyrophosphate; HMDP: hydroxymethylene diphosphonate; SPECT: single photon emission computed tomography*

At 1-h acquisition, most patients demonstrated concordant high-grade uptake of PYP and HMDP. Specifically, of the 35/36 patients graded 3 by PYP were also graded 3 by HMDP, indicating excellent agreement in cases with intense myocardial uptake (Fig. 1). Minor discrepancies were observed in the lower grades; a few patients who scored 1 or 2 on the PYP were graded higher on the HMDP. However, the overall agreement was substantial with a Cohen’s kappa value of 0.624. When applying the clinically established visual cutoff of grade ≥ 2 among the 48 patients who underwent both tracers at 1 h (45 ATTR-CA), sensitivity, specificity, and diagnostic accuracy were 97.7, 25.0, and 91.7% for PYP, and 97.8, 66.7, and 95.8% for HMDP, respectively.

At the 3-h acquisition, concordance remained high. Among the 46 patients graded as 3 by the PYP, 44 were also scored as 3 by the HMDP, with only two downgraded to grade 2. As with the 1-h acquisitions, discrepancies were more frequent in cases with mild-to-moderate uptake (grades 0–2), for which the HMDP occasionally yielded higher scores. The overall agreement was substantial with a Cohen’s kappa value of 0.630. Using the same visual cutoff of grade ≥ 2 at 3 h, sensitivity, specificity, and diagnostic accuracy were 89.6, 66.7, and 88.0% for PYP, and 85.7, 83.3, and 85.5% for HMDP, respectively.

Overall, both tracers showed a strong agreement in terms of visual grading, particularly in advanced cases (grade 3), whereas mild-to-moderate uptake demonstrated some variation between the tracers.

### Planar imaging: semiquantitative analysis (Table 2)

**Table 2:** Comparison of the semi-quantitative heart-to-contralateral lung (H/CL) ratios between PYP and HMDP

Time point	HMDP H/CL median (IQR)	PYP H/CL Median (IQR)	*p* value	Correlation coefficient
1 h	1.82 (1.61–2.09)	1.77 (1.61–2.00)	0.03	Rho = 0.839 (*p* < 0.001)
3 h	1.75 (1.47–2.16)	1.67 (1.49–1.94)	0.002	Rho = 0.905 (*p* < 0.001)

Values are shown for 1-h and 3-h acquisitions. Correlation coefficients, median values, and interquartile ranges are presented

PYP: pyrophosphate; HMDP: hydroxymethylene diphosphonate

H/CL: heart-to-contralateral lung; PYP: ^99m^Tc-pyrophosphate; HMDP: ^99m^Tc-hydroxymethylene diphosphonate; IQR: interquartile range

**Fig. 2:**
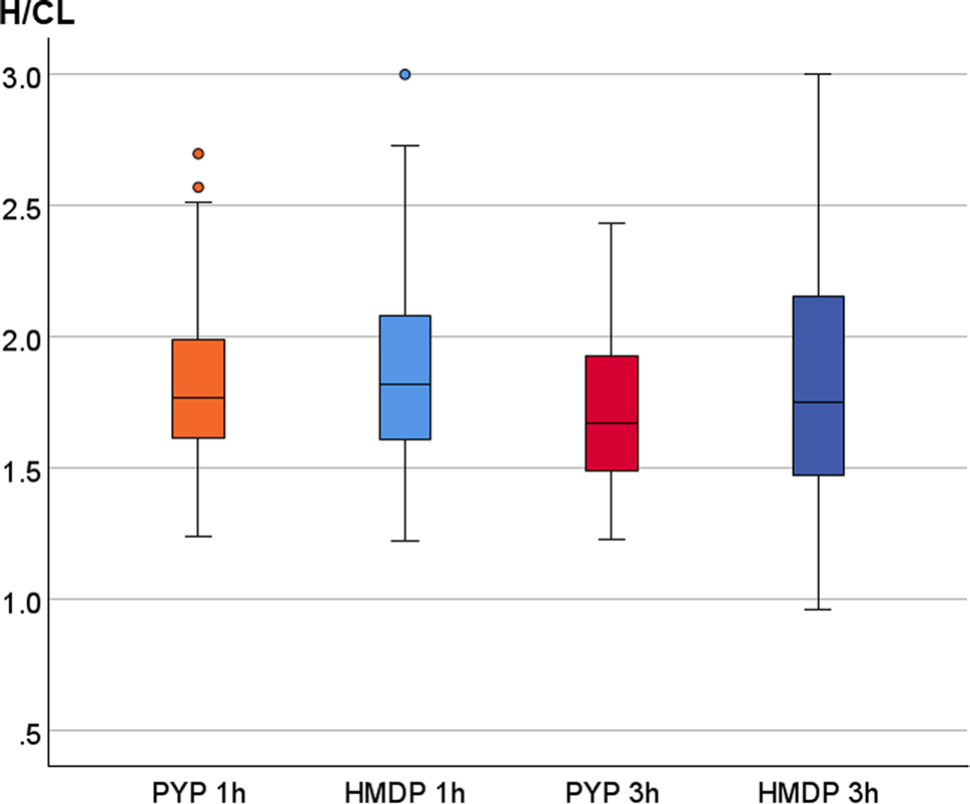
Box plots comparing the calculated heart-to-contralateral lung (H/CL) ratios between ^99m^Tc-PYP and ^99m^Tc-HMDP scintigraphy at 1-h and 3-h acquisitions. Median values, interquartile ranges, and outliers are presented. *PYP: pyrophosphate; HMDP: hydroxymethylene diphosphonate*

A direct comparison of the H/CL ratios between PYP and HMDP demonstrated close agreement for both early and delayed acquisitions. At 1 h, the median H/CL ratios were 1.77 for PYP and 1.82 for HMDP, while at 3 h they were 1.67 and 1.75, respectively (Fig. 2). HMDP tended to yield slightly higher values during delayed imaging.

**Fig. 3:**
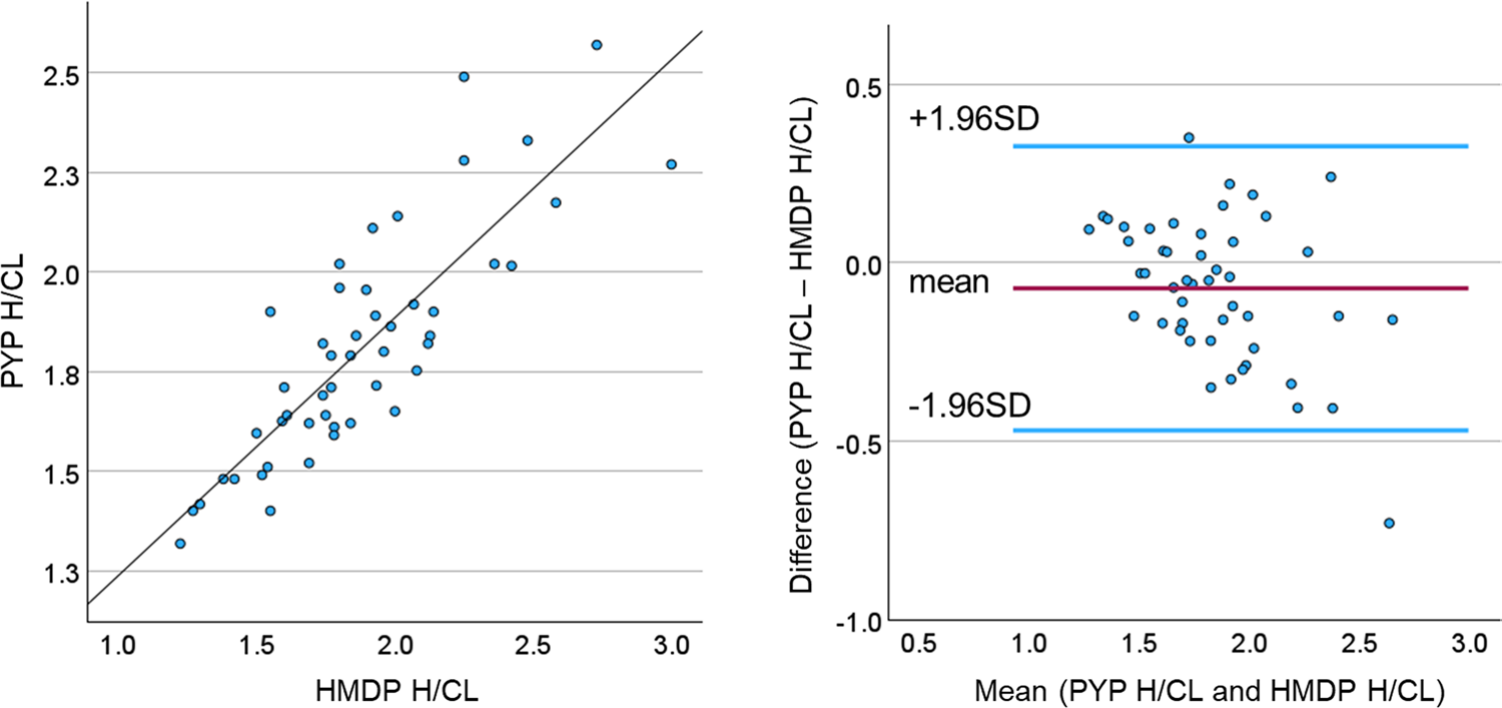
Correlation analysis and Bland–Altman plots for H/CL ratios obtained with PYP and HMDP at 1 h. The scatter plot demonstrates the linear relationship between tracers, and the Bland–Altman plot illustrates the fixed and proportional biases. *PYP: pyrophosphate; HMDP: hydroxymethylene diphosphonate*

**Fig. 4:**
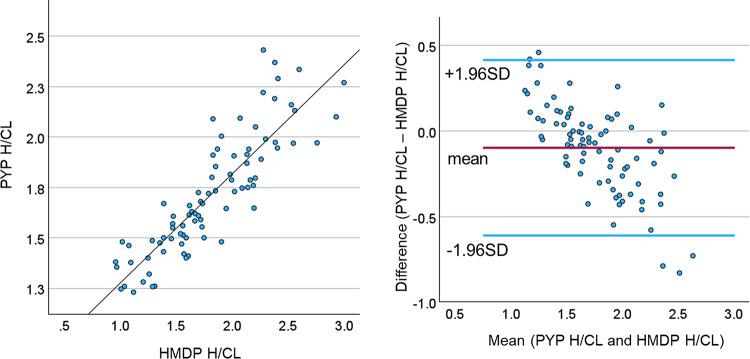
Correlation analysis and Bland–Altman plots for the H/CL ratios obtained with PYP and HMDP at 3 h. A strong correlation was observed, with systematic differences noted in Bland–Altman analysis. *H/CL: Heart-to-contralateral lung; PYP: pyrophosphate; HMDP: hydroxymethylene diphosphonate*

Correlation analysis revealed strong associations between PYP and HMDP, with Spearman’s rho values of 0.839 at 1 h and 0.905 at 3 h (both *p* < 0.001) (Fig. 3 and Fig. 4).

Bland–Altman analysis revealed systematic differences between the two tracers. At both the 1-h and 3-h acquisitions, there was evidence of both fixed and proportional bias, indicating that the discrepancy between tracers increased with higher H/CL ratios.

Using established H/CL cutoff values (≥ 1.5 at 1 h and ≥ 1.3 at delayed imaging), both tracers demonstrated high diagnostic efficacy. At 1 h, the sensitivity, specificity, and diagnostic accuracy were 88.6, 75.0, and 87.5% for PYP, and 95.6, 66.7, and 93.8%, respectively. At 3 h, PYP demonstrated a sensitivity, specificity, and diagnostic accuracy of 97.4, 33.3, and 92.8%, respectively, whereas HMDP showed 89.6, 66.7, and 88.0%, respectively.

Overall, these findings indicate that PYP and HMDP provide comparable semi-quantitative information, although HMDP tends to produce slightly higher delayed values and systematic differences exist across the measurement range.

### SPECT imaging: visual analysis

Visual assessment was performed using the standard cutoff of grade ≥ 2. Using this criterion, both PYP and HMDP demonstrated identical diagnostic performances. The sensitivity, specificity, and diagnostic accuracy of both tracers were 98.7, 83.3, and 97.6%, respectively. When patients with AL amyloidosis were excluded from the analysis, the diagnostic accuracy of both tracers was 100%. These findings indicate the comparable diagnostic efficacies of PYP and HMDP in SPECT imaging when clinically established visual criteria are applied.

### SPECT imaging: semiquantitative analysis (Table 3)

**Table 3:** Results of SPECT-based relative segmental uptake (RSU) analysis comparing PYP and HMDP across the 17-segment model. Median RSU values and IQR for each myocardial segment are presented

Segment AHA 17 model	PYP RSU median (IQR)	HMDP RSU median (IQR)
1	0.96 (0.92–1.00)	0.96 (0.90–0.99)
2	1.03 (0.96–1.10)	1.06 (1.00–1.14)
3	1.05 (1.00–1.11)	1.10 (1.04–1.18)
4	1.04 (1.01–1.10)	1.10 (1.04–1.13)
5	1.05 (1.00–1.12)	1.03 (0.97–1.11)
6	0.96 (0.93–1.02)	0.95 (0.90–1.00)
7	0.94 (0.89–0.98)	0.91 (0.87–0.95)
8	1.03 (0.98–1.08)	1.09 (1.00–1.15)
9	1.12 (1.07–1.20)	1.21 (1.14–1.27)
10	1.09 (1.05–1.12)	1.08 (1.03–1.13)
11	1.02 (0.97–1.04)	1.00 (0.95–1.03)
12	0.97 (0.91–0.99)	0.95 (0.87–0.98)
13	0.90 (0.81–0.94)	0.84 (0.78–0.90)
14	1.03 (0.98–1.09)	1.04 (0.96–1.10)
15	1.02 (0.99–1.05)	1.00 (0.97–1.05)
16	0.92 (0.85–0.97)	0.86 (0.81–0.94)
17	0.89 (0.84–0.94)	0.83 (0.77–0.88)

PYP: pyrophosphate; HMDP: hydroxymethylene diphosphonate; IQR: Interquartile range

SPECT: single photon emission computed tomography; RSU: relative segmental uptake; PYP: ^99m^Tc-pyrophosphate; HMDP: ^99m^Tc-hydroxymethylene diphosphonate; AHA: American Heart Association; IQR: interquartile range

**Fig. 5:**
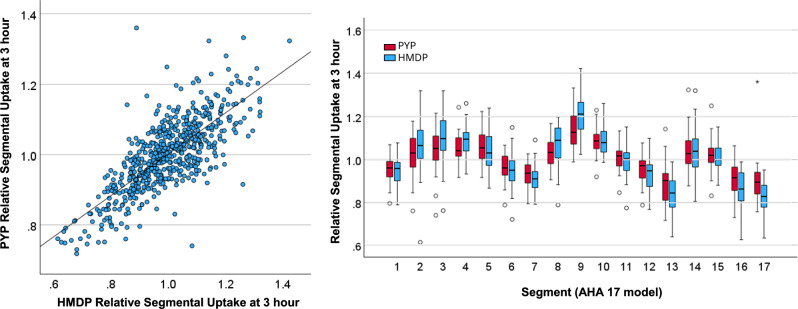
SPECT-based relative segmental uptake (RSU) analysis. Left: correlation of RSU values between PYP and HMDP across 17 segments. Right: multiple line plots illustrating segmental RSU values in each myocardial region, demonstrating consistent basal-to-apical gradients for both tracers. *SPECT: single photon emission computed tomography; PYP: pyrophosphate; HMDP: hydroxymethylene diphosphonate*

Quantitative analysis using the QPS software was feasible for 34 patients in whom the RSU values were obtained for each of the 17 myocardial segments defined by the AHA model. Correlation analysis further demonstrated a significant association between tracers. The segment-based RSU values for PYP and HMDP showed a Spearman’s correlation coefficient of 0.744 (*p* < 0.001), indicating a substantial agreement across the entire left ventricle (Fig. 5).

The segmental distribution patterns of uptake were similar between the tracers, both of which demonstrated relatively higher RSU in the basal to mid-septal segments and inferior wall, whereas lower uptake was observed in the apical segments. The mean RSU curves for the PYP and HMDP exhibited nearly overlapping profiles, with only minor differences in some segments.

Overall, the SPECT analysis confirmed that PYP and HMDP provided highly consistent regional uptake patterns, supporting their comparability in semiquantitative myocardial assessments.

### ROC curve analysis

ROC curve analysis demonstrated good diagnostic performance of the H/CL ratios for differentiating ATTR-CA from non-ATTR cases using both tracers.

**Fig. 6:**
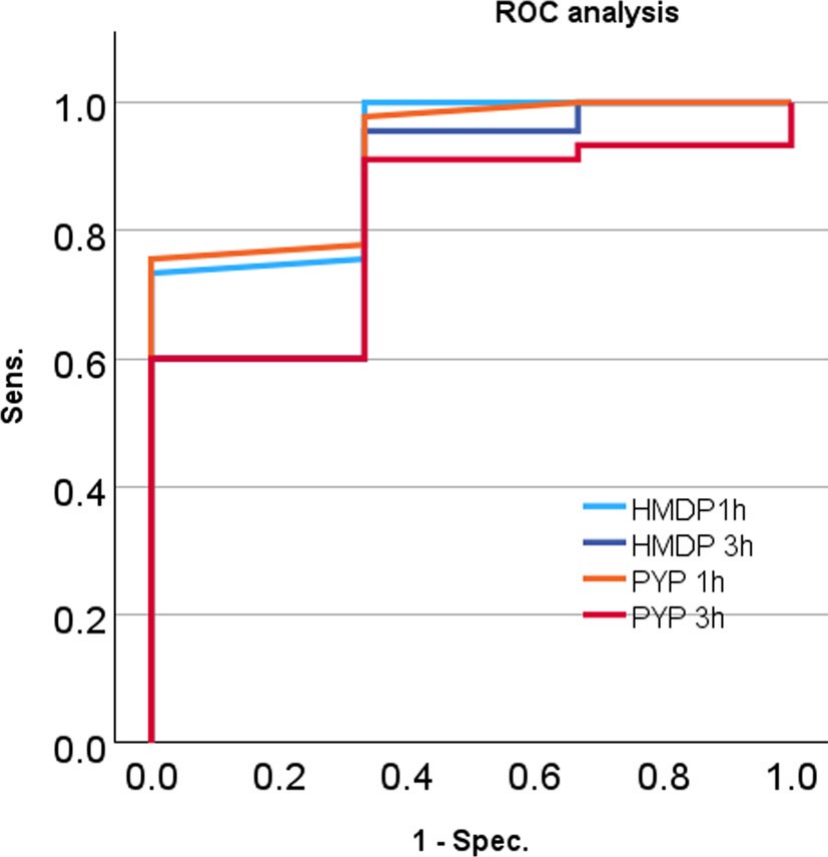
Receiver operating characteristic (ROC) curves comparing the diagnostic performance of PYP and HMDP. ROC curve analyses for 1-h and 3-h acquisitions are shown, with area under the curve (AUC) values indicating excellent accuracy at 1 h and good accuracy at 3 h. *PYP: pyrophosphate; HMDP: hydroxymethylene diphosphonate*

At 1 h, the AUC for PYP and HMDP were 0.919 and 0.915, respectively. After 3 h, the AUC were slightly lower (0.815 for PYP and 0.852 for HMDP; Fig. 6). Both the tracers showed excellent discrimination in early imaging and moderate-to-good discrimination in delayed imaging.

A direct comparison of the paired AUCs revealed small but significant differences between certain conditions. Specifically, the AUC at 1 h was significantly higher than that at 3 h for both tracers (PYP 1 h vs PYP 3 h: ΔAUC = 0.104, *p * = 0.014; HMDP 1 h vs HMDP 3 h: ΔAUC = 0.063, *p* = 0.036). However, no significant differences were observed between the PYP and HMDP groups at these time points (1 h vs. 1 h, *P* = 0.865; PYP 3 h vs. HMDP 3 h, *P* = 0.343).

## Discussion

In this head-to-head study, we directly compared PYP and HMDP scintigraphy findings in a patient cohort that underwent both examinations. Both tracers showed high and broadly comparable diagnostic performance for suspected cardiac amyloidosis. Visual analysis further demonstrated substantial agreement (κ = 0.624 at 1 h, κ = 0.630 at 3 h), particularly in cases with grade 3 uptake. Semi-quantitative H/CL ratios were strongly correlated between the tracers at both time points, and ROC curve analyses confirmed excellent diagnostic accuracy at 1 h (AUC > 0.90) and good accuracy at 3 h (AUC > 0.80). Bland–Altman analysis further revealed fixed and proportional biases, indicating systematic differences that became more pronounced at higher uptake levels.

These findings are consistent with those of previous studies. Treglia et al. previously reported a pooled sensitivity and specificity > 90% for bone-avid tracers, including PYP, DPD, and HMDP, in the diagnosis of ATTR-CA [12]. Ahluwalia et al. confirmed these results in a more recent meta-analysis, demonstrating excellent diagnostic accuracy across tracers and highlighting subtle differences in uptake characteristics [13]. Wu and Yu further extended this evidence in a multimodal meta-analysis, showing that bone-avid tracer SPECT achieved outstanding diagnostic performance, and subgroup analysis additionally suggested that HMDP provided the highest sensitivity and PYP the highest specificity, with all tracers achieving an AUC value of 0.99 [14]. Aldehlaui et al. added further rigor by restricting the analysis to biopsy-proven cases and reported that HMDP achieved slightly higher sensitivity and specificity than PYP, reinforcing the concept that small but systematic differences exist between tracers despite their excellent overall accuracy [15].

In addition to these meta-analyses, Park et al. directly compared DPD and PYP scintigraphy in patients with cardiac amyloidosis, showing that DPD uptake correlated more strongly with clinical variables, such as renal function, natriuretic peptides, and echocardiographic indices, while PYP tended to yield higher visual scores and to identify more patients with grade ≥ 2 uptake [16]. Although limited by a relatively small sample size, these results underscore the fact that tracers differ in their uptake kinetics and reflect disease severity. Our results for PYP and HMDP paralleled these findings, as both tracers correlated strongly, yet systematic biases were present, and HMDP demonstrated a slightly higher delayed uptake.

Our additional analyses using clinically established cutoffs demonstrated that PYP and HMDP have high and comparable diagnostic performances for ATTR-CA across planar and SPECT imaging. In particular, the visual assessment of SPECT achieved near-perfect diagnostic performance, reaching 100% accuracy after the exclusion of AL amyloidosis, supporting the robustness of SPECT-based evaluation within the current non-biopsy diagnostic framework. The differences between the planar and SPECT-based visual scores observed in our study are consistent with those of previous reports and likely reflect the superior ability of SPECT/CT to distinguish true myocardial uptake from blood pool activity and overlapping structures [17]. Accordingly, recent reports and expert recommendations increasingly advocate SPECT/CT-based interpretation to improve diagnostic reliability in routine clinical practice [10, 17]. The relatively low specificity observed in some planar analyses should be interpreted cautiously, as it is likely influenced by the limited number of non-ATTR-CA cases in our cohort, rather than true tracer-related differences.

Importantly, Sperry et al. analyzed PYP uptake using the AHA 17-segment model, and identified a characteristic regional distribution, with greater uptake in the basal and mid-ventricular segments compared with the apex, a pattern consistent with “apical sparing.” [18] These authors also defined an apical-sparing ratio that was significantly associated with mortality, whereas global indices such as H/CL were not predictive. Our RSU analysis further showed that PYP and HMDP exhibited similar segmental distributions with relatively higher uptake in the basal and septal segments. This concordance across tracers supports the robustness of regional uptake patterns and suggests that apical sparing, a hallmark finding of PYP, is equally demonstrable with HMDP. Thus, the diagnostic and potential prognostic implications of segmental uptake patterns may be generalizable across different tracers.

Overall, both tracers demonstrated high diagnostic performances; however, subtle differences were observed. PYP is the most extensively validated bone-avid tracer worldwide, has been supported by numerous studies, and has been incorporated into major international guidelines. This factor performed particularly well at 1-h imaging in our cohort, suggesting that early acquisition protocols could be reliably applied in clinical practice. However, delayed imaging showed slightly lower H/CL values than HMDP, and uptake in mild-to-moderate cases was occasionally underestimated. In contrast, HDMP tends to yield higher delayed H/CL ratios and has been reported to produce lower blood-pool activity, which could enhance the myocardial-to-background contrast. Overall, these features could improve diagnostic confidence in borderline cases. At the same time, our Bland–Altman analysis demonstrated proportional bias, with discrepancies becoming more evident at higher uptake levels. Moreover, although HMDP has been widely applied in Europe and Japan, it has been less extensively validated in other regions, and standardized diagnostic thresholds remain less firmly established compared with PYP.

This study have some limitations. The retrospective single-center study design led to the inclusion of relatively few non-ATTR cases. In addition, interobserver reproducibility was not assessed. Bland–Altman analysis indicated that absolute H/CL values were not interchangeable, underscoring the need for multicenter harmonization and tracer-specific thresholds. It should also be noted that, in contemporary clinical practice, H/CL ratios are regarded as supportive semi-quantitative metrics rather than absolute diagnostic criteria, with diagnosis primarily based on integrated visual assessment and exclusion of AL amyloidosis [10].

Furthermore, segment-based RSU analysis using QPS is not a standardized quantitative method for bone-avid tracer imaging, and reliable LV segmentation may be challenging in cases with low-grade myocardial uptake; therefore, RSU analysis was restricted to technically adequate cases. Blood-pool–based normalization was not performed and may provide complementary information in future studies. Future studies should further evaluate segmental uptake differences between tracers, validate apical-sparing indices using HMDP, and assess their prognostic implications.

## Conclusion

Both PYP and HMDP have demonstrated excellent diagnostic accuracy for cardiac amyloidosis. PYP is well established worldwide but shows slightly higher delayed uptake and favorable background characteristics. Importantly, diagnostic performance on SPECT imaging was comparable between PYP and HMDP, underscoring their equivalent utility when applied within contemporary non-biopsy diagnostic algorithms. In addition, regional uptake patterns such as apical sparing appear to be conserved across tracers, indicating that both can reliably characterize disease distribution. The standardization of acquisition and interpretation protocols is essential to ensure diagnostic consistency and enable broader clinical applications.

## Abbreviations

AHA: American heart association
AL: Immunoglobulin light-chain
AL-CA: Immunoglobulin light-chain cardiac amyloidosis
ASNC: American society of nuclear cardiology
ATTR: Transthyretin
ATTR-CA: Transthyretin cardiac amyloidosis
AUC: Areas under the curve
CA: Cardiac amyloidosis
DPD: 3,3-Diphosphono-1,2-propanodicarboxylic acid
HMDP: Hydroxymethylene diphosphonate
H/CL: Heart-to-contralateral lung
JCS: Japanese Circulation Society
LEHR: Low-energy high-resolution
PYP: Pyrophosphate
QPS: Quantitative perfusion SPECT
RSU: Relative segmental uptake
ROC: Receiver operating characteristic
